# Editorial: Nanotoxicology and Environmental Risk Assessment of Engineered Nanomaterials (ENMs) in Plants

**DOI:** 10.3389/fpls.2016.01370

**Published:** 2016-09-12

**Authors:** Nelson Marmiroli, Jason C. White

**Affiliations:** ^1^Department of Life Sciences, University of ParmaParma, Italy; ^2^Department of Analytical Chemistry, Connecticut Agricultural Experiment StationNew Haven, CT, USA

**Keywords:** environmental health and safety, food safety, nanotoxicity, phytotoxicity, risk assessment, nanotoxicology

In the last two decades, the development and application of nanotechnology—the science of small—has increased dramatically, impacting sectors as far reaching as medicine (including disease treatment and diagnosis), energy, communications, water treatment and food production. In spite of these incredible advances, the general consensus among the scientific community is that our understanding of the fate and effects of these novels materials in the environment is inadequate. Given the level of current and projected exposure to both human and non-human receptors, this basic lack of understanding with regard to ENM environmental health and safety (EHS) is highly disconcerting.

The articles contributed as part of this research topic are indicative of the current work being done to begin to address some of these key knowledge gaps, specifically focusing on impacts of ENM exposure to terrestrial plant species and food crops. The papers address a range of topics, including effects on key plant species measured at both the physiological and molecular level, and present a description of some important analytical platforms by which to assess exposure; single particle inductively coupled mass spectrometry (sp-ICP-MS) and proteomics. Two articles specifically address ENM impacts on foods nutritional content and on dietary intake. A final review article assesses the current use of carbon nanomaterials in agriculture, including a description of key research areas where more work is sorely needed.

Specifically, Mukherjee et al. investigated the role of coating type on the toxicity and accumulation of ZnO nanoparticles (NP) to soil-grown green pea (*Pisum sativum* L.). Importantly, this work as conducted to analyze the impact of exposure during the full life cycle of the plant and included a direct comparison of the nanoparticles against corresponding bulk and ion controls. The authors report that exposure to the nanoparticle form uniquely impacted Zn levels in the plant, as well as photosynthetic pigments but that protein and carbohydrate profiles were largely unaffected. However, among the nanoparticle types, overall phytotoxicity and seed nutritional quality was impacted by particle coating.

Mattiello et al. investigated the phytotoxic and genotoxic effects of CeO_2_ and TiO_2_ NP exposure on barley under hydroponic conditions. The authors measured genotoxicity by Randomly Amplified Polymorphism DNA and by the mitotic index. The authors reported greater overall toxicity from CeO_2_ as compared to TiO_2_. The authors also observed changes in RAPD banding and in the mitotic index upon NP exposure, as well as NP-specific effects on cellular oxidative status and ATP levels. Importantly, the authors also used transmission electron microscopy with energy dispersive X-ray spectroscopy to detect NP presence within the exposed root cells.

Zuverza-Mena et al. investigated the impacts of NP Ag on the physiological status and nutritional quality of radish under hydroponic conditions. The authors reported that although seed germination was unaffected by Ag exposure, water content and both root and shoot development were negatively impacted. Importantly, the content of several important nutrients and biomolecules were negatively impacted by Ag NP treatment, including micronutrients, lipid/protein content, and structural molecules such as lignin, pectin, and cellulose.

Ebbs et al. used data from their previous studies on NP oxides and ions of Zn, Cu, and Ce to investigate dietary intake through the calculation of oral reference doses in carrot. The authors used reverse dietary intake calculations to estimate the number of serving sizes, mass of carrots, and the carrot content of Zn, Cu, and Ce that would be needed to approach oral RfD values. The authors showed that peeling carrots was the most important activity for effectively reducing dose. The calculations demonstrated that exposures of concern are only likely to occur during unrealistic scenarios of excessive consumption.

Wang et al. evaluated the impact of ZnO NP on *Arabidopsis thaliana* growth and photosynthetic output. The authors reported that exposure to ZnO reduced plant biomass by up to 80% and decreased chlorophyll content by more than 50%. Carotenoid content was unaffected. The authors used quantitative RT-PCR to measure the expression of some key photosynthetic genes. The overall phytotoxicity was linked to inhibition of the expression of chlorophyll synthesis and photosystem genes.

Marmiroli et al. investigated the impacts of CdS quantum dot exposure on wild type and mutant (tolerant) *Arabidopsis*, correlating proteomic and transcriptomic responses with regard to tolerance and toxicity. The authors report a strong correlation between proteomic and transcriptomic endpoints, although a notable number of gene responses seemed to differ when assessed at the transcript vs. protein level. The authors discuss likely causes for this discrepancy, as well as the potential use of this type of “–omic” approach in ENM effect studies.

Bao et al. used a combination of TEM and sp-ICP-MS to characterize Ag NP accumulation within exposed *Arabidopsis*. The authors validated a digestion protocol that could release Ag NPs without significantly altering particle size. Root and shoot Ag NP data from sp-ICP-MS correlated well with results obtained via electron microscopy.

Mukherjee et al. provide a thorough review of the use of carbon nanomaterials (CMN) in agriculture. The authors discuss the seemingly contradictory findings in the literature, with roughly equal numbers of studies demonstrating enhancement effects on plant health and performance as a function of CNM exposure as compared to the large number of papers showing phytotoxicity as measured by a range of parameters. The authors discuss the effect of CNM exposure on co-contaminant effects. Last, the authors highlight critical knowledge gaps in this area and discuss the overall need for more mechanistic investigations focused on plant-CNM interactions so as to better characterize exposure and risk.

This research topic addresses many of the key knowledge gaps that exist with regard to nanomaterial impacts on terrestrial plants. The individual contributions focus not only on measuring effects at the physiological and molecular levels but also address key topics of novel particle detection platforms and also on overall impacts on human nutrition and dietary intake. Although the work in this compilation of papers is impressive, perhaps the most consistent theme through these studies is the great need for more intensive investigation into nanomaterial fate and effects in agriculture and food production. Only then will a robust and accurate assessment of exposure and risk be possible.

## Author contributions

All authors listed, have made substantial, direct and intellectual contribution to the work, and approved it for publication.

### Conflict of interest statement

The authors declare that the research was conducted in the absence of any commercial or financial relationships that could be construed as a potential conflict of interest.

